# Implementing the Child Psychosis-risk Screening System in Pediatric-to-Psychiatric Care: A Novel Approach Using Child Behavior Checklist Data

**DOI:** 10.1192/j.eurpsy.2025.1141

**Published:** 2025-08-26

**Authors:** Y. Hamasaki, Y. Sakaue, S. Michikoshi, T. Nakayama, S. Ueba, M. Isobe, T. Hikida

**Affiliations:** 1 Faculty of Contemporary Society, Kyoto Women’s University, Kyoto; 2 Department of Pediatrics, Shiga University of Medical Science, Shiga; 3 Faculty of Data Science, Kyoto Women’s University, Kyoto; 4 Department of Pediatrics, Saiseikai Moriyama Municipal Hospital, Shiga; 5 Department of Psychiatry, Kyoto University Graduate School of Medicine, Kyoto; 6 Laboratory for Advanced Brain Functions, Osaka University, Osaka, Japan

## Abstract

**Introduction:**

Screening children for the potential development of psychosis is challenging, resulting in a suboptimal healthcare transition from pediatrics to psychiatry.

**Objectives:**

In this prospective study, we aimed to evaluate the predictive ability of the Child Psychosis-Risk Screening System (CPSS) for schizophrenia spectrum disorder (SSD) by observing the outcomes of pediatric and psychiatric outpatients.

**Methods:**

A total of 478 outpatients aged 6–18 years visiting the pediatric and psychiatric departments of university and community hospitals were enrolled in this study. Assessments included the Child Behavior Checklist (CBCL) and clinical data (sex, age, birth month, chief complaint, diagnosis, abuse, bullying, and withdrawal). The CPSS calculated the risk of developing SSD using eight CBCL subscale scores. The presence of SSD was confirmed after one year. Receiver operating characteristic (ROC) curve analysis was used to evaluate the accuracy of the CPSS in predicting SSD onset. Light-gradient boosting machine (LightGBM) learning algorithm was used to calculate the importance of each clinical data point for SSD onset prediction.

**Results:**

ROC analysis demonstrated that CPSS showed adequate predictive power for determining SSD onset (area under the curve = 0.902, 95% confidence interval: 0.866–0.939). LightGBM revealed that the importance of the CPSS risk% in predicting SSD onset exceeded other variables, including the CBCL Thought Problems subscale.

**Conclusions:**

The CPSS demonstrated adequate predictive power for SSD development and may serve as an objective adjunctive diagnostic method to screen children at risk for SSD who require early psychiatric referral. As a simple screening system utilizing common clinical practice CBCL data, we advocate CPSS implementation in pediatric-to-psychiatric healthcare.

**Disclosure of Interest:**

None Declared

